# Deconstructing the functional neuroanatomy of the choroid plexus: an ontogenetic perspective for studying neurodevelopmental and neuropsychiatric disorders

**DOI:** 10.1038/s41380-022-01623-6

**Published:** 2022-05-26

**Authors:** Byron K. Y. Bitanihirwe, Paulo Lizano, Tsung-Ung W. Woo

**Affiliations:** 1 Humanitarian and Conflict Response Institute, University of Manchester, Manchester, UK; 2 Department of Psychiatry, Harvard Medical School, Boston, MA USA; 3 Department of Psychiatry, Beth Israel Deaconess Medical Center, Boston, MA USA; 4 Translational Neuroscience Division, Beth Israel Deaconess Medical Center, Boston, MA USA; 5 Program in Molecular Neuropathology, McLean Hospital, Belmont, MA USA

**Keywords:** Neuroscience, Schizophrenia

## Abstract

The choroid plexus (CP) is a delicate and highly vascularized structure in the brain comprised of a dense network of fenestrated capillary loops that help in the synthesis, secretion and circulation of cerebrospinal fluid (CSF). This unique neuroanatomical structure is comprised of arachnoid villi stemming from frond-like surface projections—that protrude into the lumen of the four cerebral ventricles—providing a key source of nutrients to the brain parenchyma in addition to serving as a ‘sink’ for central nervous system metabolic waste. In fact, the functions of the CP are often described as being analogous to those of the liver and kidney. Beyond forming a barrier/interface between the blood and CSF compartments, the CP has been identified as a modulator of leukocyte trafficking, inflammation, cognition, circadian rhythm and the gut brain-axis. In recent years, advances in molecular biology techniques and neuroimaging along with the use of sophisticated animal models have played an integral role in shaping our understanding of how the CP–CSF system changes in relation to the maturation of neural circuits during critical periods of brain development. In this article we provide an ontogenetic perspective of the CP and review the experimental evidence implicating this structure in the pathophysiology of neurodevelopmental and neuropsychiatric disorders.

## Introduction

The choroid plexus (CP) was first discovered by Herophilus (*c335-c280 BC*), a Greek physician and anatomist [1, 2]. Since Herophilus’ seminal observations, modern neuroanatomy advancements have led to the CP being appreciated as a network of capillaries and specialized monolayer of cuboidal epithelial cells (ependymal cells) [3, 4]. CPs are located in the four ventricles of the brain and are the principal source of cerebrospinal fluid (CSF)—an interstitial fluid (ISF) that plays a key role in brain physiology [5, 6]. Beyond modulating brain buoyancy [7], intracranial pressure [8], inflammatory signalling [9, 10] and ionic homeostasis [8, 11], the CSF also serves as a source of nutrients [12] and hormones [13]—through volume transmission [14]—in addition to functioning as a toxin barrier for the brain [15].

Numerous neural mechanisms can modulate CSF production and choroidal blood flow [16]. Because the CP-CSF system plays an integral role in brain development and neurophysiological processes—such as neurogenesis [17], circadian rhythms [18, 19], neural circuit plasticity [20] and gut microbiota-immune interactions [21], which in turn modulate cognitive/behavioural processes [2]—it follows that any damage to the CP could lead to a wide spectrum of brain diseases [22]. Thus, the present article provides an ontogenetic perspective focusing on the CP as well as providing in vitro and in vivo experimental evidence implicating the CP in neurodevelopmental and neuropsychiatric conditions.

## Neuroanatomy of the Choroid Plexus

**Fig. 1:**
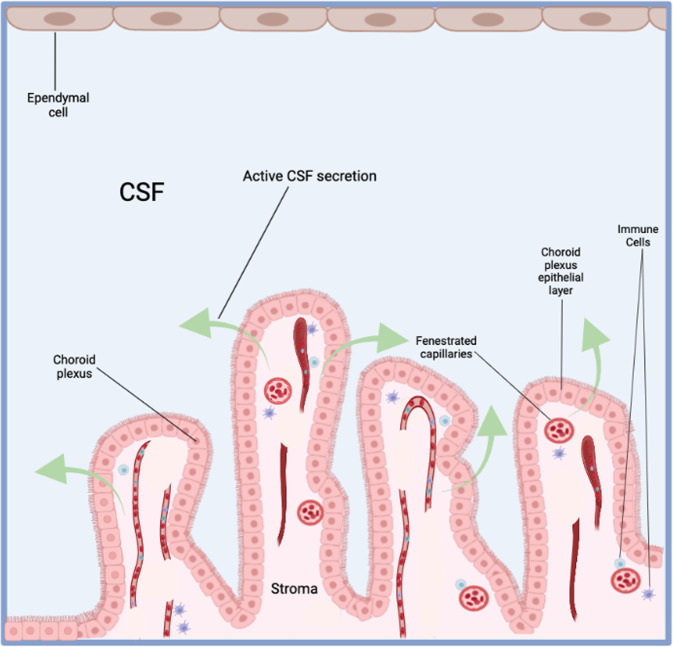
Neuroanatomy of the choroid plexus.

The CPs have a central stroma covered by a single epithelial layer; the choroid plexus epithelium (CPE) (Fig. 1). The stroma is highly vascularized, consisting of connective tissue, pericytes and blood vessels [3]. These blood vessels are fenestrated (60-80 nm openings) and leaky, making them distinct from cerebral penetrating blood vessels which are connected by tight junctions (comprised of occludins, claudins and adhesion molecules) and form the blood-brain barrier (BBB) [3, 6]. The Blood-CSF barrier is highly restrictive and regulates the passage of select molecules, ions, and cells (via an array of transport, physical and metabolic machineries) between the blood and the central nervous system (CNS) [23, 24].

A diversity of ion channels, pumps and transporters are expressed at the basolateral and apical membranes of the CPE cells that modulate the net flux of sodium, chloride, and bicarbonate ions across the CPE from the blood-to-CSF side [25]. This generates an osmotic gradient that drives plasma water across the CP membranes into the ventricles [26]. Conversely, there is a net flux of potassium from the CSF-to-blood [25]. Notably, CPE cells display morphological diversity with the apical membranes exhibiting microvilli along with primary and motile cilia, while the basolateral membranes form an ‘interdigitating’ labyrinth with adjacent cells at the transition zone from basal to lateral domains [3, 27]. Motile cilia are integral for CSF circulation and play a key role in sensory function related to mechanical loading, shear stress, osmotic force and fluid flow with a disruption in motile ciliary beating contributing to ventricular defects [28].

## Ontogenesis of the choroid plexus

Choroid plexus development begins at week 7 of gestation with mesenchymal cells invaginating into the neural tube at the sites of cerebral ventricles formation, starting with the fourth, lateral, and then third ventricles [22, 29]. The process of CP development is divided into four stages depending on the morphological and histological changes that occur within the cells [30–32] (Table 1). Stage 1 occurs at week 7 of gestation with pseudostratified epithelial cells that lack glycogen and identifiable villi. Stage 2 starts at week 9 with glycogen-laden short columnar cells consisting of sparse microvilli with apically located nuclei. Stage 3 takes place at week 17 with most epithelial cells having adopted a cuboidal structure bearing microvilli from the apical surface and exhibit moderate levels of glycogen with apical and central nuclei. Stage 4 is the final stage, which commences at week 29 and consists of cuboidal epithelial cells with basal or central nuclei and an absence of glycogen. The role of glycogen during CP development remains unknown with some positing its involvement in the formation of a glycogen rich basement membrane or it may serve a nutritive function [30]. Notably, high levels of carbonic anhydrase are detected in the developing CP which persist through structural maturity [31]. Carbonic anhydrase is central for CSF synthesis via the formation of bicarbonate ions, that are transported across the CPE via an apical sodium/potassium pump. Also, during development, immature CP cells contain a diversity of plasma proteins including transferrin, α-fetoprotein, transthyretin, albumin and transferrin. However, only transthyretin is detected in mature CP cells [6].

**Table 1:** Stages of differentiation of the human choroid plexus according to Netsky and Shuangshoti [136].

	Stage 1	Stage 2	Stage 3	Stage 4
Time of development	7th Week	9th Week	17th week	29th week
Duration of development	2 Weeks	8 weeks	12 weeks	11 weeks
Epithelium	Pseudo-stratified, tall, central nuclei	Low columnar, apical nuclei	Cuboidal, apical and central nuclei	Cuboid or squamous, central and basal nuclei
Glycogen	Absent	Abundant	Moderate	Minimal and absent
Villi	Absent	Sparse	Primary villi	Villi with multiple fronds
Stroma	Loose mesenchyme	Small numbers of connective fibres	Moderate numbers of fibres	Large numbers of fibres and meningocytes
Blood Vessels	Ill-defined vascular walls	Defined vascular walls	Well-formed vascular walls	Same
Size in relation to ventricle	Tiny	Extremely large	Moderately large	Small

Although little is known about the molecular mechanisms directing fenestrated vessel formation in the CP, a recent study using zebrafish larval CP and CPEs demonstrated ultrastructural conservation of the CPs across vertebrate species [32]. Specifically, this study identified a combination of vascular endothelial growth factors required for fenestrated vessel formation [32]. Other studies have started to clarify the expression patterns involved in CP development, with recent genetic experiments in the developing mouse brain revealing distinct splicing signatures and expression programs in regulating CPE development [5, 33, 34].

## Physiological functions of the choroid plexus

The CP serves a diversity of physiological functions in the CNS ranging from CSF production, neural stem cell behaviour and immune cell trafficking to modulating the microbiome gut-brain axis, circadian rhythms and cognition (see Table 2). In this section we discuss CP function.

**Table 2:** Summary of functions of the CP in the central nervous system.

Function influenced by CP	References
Cognitive functions	
Learning and memory	[137]
Anxiety	[61]
PTSD-like behaviour	[64]
Depression	[138]
Neurophysiological/cell biological functions	
Critical period regulation and neural circuit plasticity	[139, 140]
Chemical surveillance	[141, 142]
CSF production	[6]
Circadian rhythms	[18, 143]
Ion homeostasis and intercellular transport of molecules	[25]
Neuroprotection	[144]
Gut microbiota–immune interactions	[21]
Neurogenesis	[57, 59]
Inflammatory signalling	[64, 65, 145]
Stress response	[64]

*CP* choroid plexus, *CSF* cerebrospinal Fluid, *PTSD* post-traumatic stress disorder.

### Cerebrospinal fluid production and the glymphatic system

Choroid plexus epithelial cells are essential for CSF production and secrete between 400ml-600ml of CSF per day in the adult human brain [6]. Cerebrospinal fluid synthesis by the CP occurs in two phases: passive fluid ultrafiltration across CP capillaries followed by active transport across the CPE [11, 35]. Plasma filters through CP capillaries into the ISF, a process that depends on pressure and homeostatic regulation of ionic gradients [11]. For example, sodium, calcium, potassium, chloride and bicarbonate co-transporters/exchangers translocate these ions in an energy (adenosine triphosphate, ATP) dependent fashion from the ISF across the basolateral membrane into the CPE cell via the apical membrane [36–42]. Aquaporins (AQPs, particularly AQP1) facilitate movement of water across the CPE from plasma to ventricular lumen down an osmotic gradient, a process that regulates CSF production [43]. Notably, human studies indicate that CSF production varies during the day with magnetic resonance imaging (MRI) studies suggesting that peak production occurs just after midnight [44]. Thus, it is hypothesized that circadian mediated CSF production might be attributed to autonomic innervation of the CP [45].

The study of CP function has gained increased attention in recent years due to physiological and immunological research revealing the significance of the ‘glymphatic’ drainage system—a glial-dependent waste clearance pathway in the brain—of vertebrates with implications for vascular and fluid clearance disorders such as multiple sclerosis, Parkinson’s disease, and Alzheimer’s disease [46, 47]. A number of theories have emerged on the function and structure of the glymphatic system and associated CSF clearance pathways [12, 48]; however, a central tenet to existing glymphatic theories is that this pathway serves a central function for paravascular CSF and ISF exchange, and in coordination with meningeal lymphatic vessels, facilitates clearance of metabolic waste products, including amyloid beta, toward cervical lymph nodes [49].

### Cerebrospinal fluid circulation

**Fig. 2:**
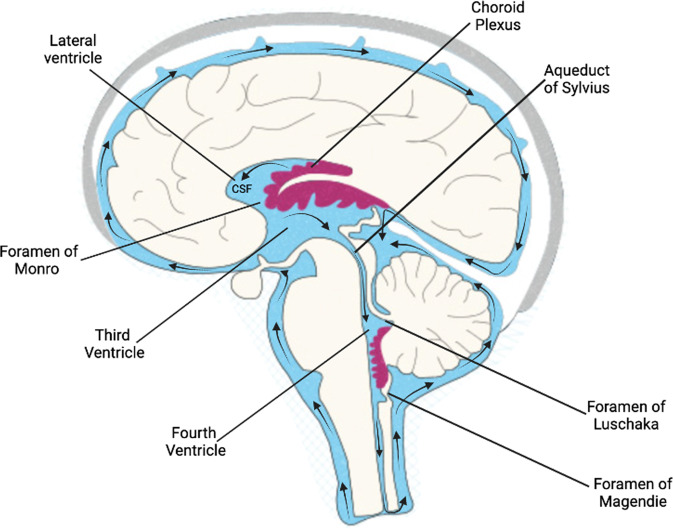
Ventricular system––pathway of cerebrospinal fluid flow.

Current models of CSF dynamics and circulation from animal intracranial pressure measurements and from human phase-contrast MRI indicate that the CSF flows in a craniocaudal direction from the lateral ventricles to the third ventricle via bilateral interventricular foramen of Monro [8, 50]. Cerebrospinal fluid then flows to the fourth ventricle via the cerebral aqueduct of Sylvius. Lastly, CSF flows to the subarachnoid space via the foramen of Magendie medially and foramen of Luschka laterally [1] (Fig. 2). Once in the subarachnoid space, CSF is reabsorbed through the arachnoid granulations that transport it into the dural venous sinuses, channelling the CSF back into the venous circulation. Although intracranial arterial pulsations are thought to drive CSF flow [51], recent evidence suggests that respiration and venous pressure represent a new mechanism for CSF circulation [52]. Cerebrospinal fluid pressure can alter brain development; too little CSF can stunt brain growth whereas overproduction of CSF can lead to hydrocephalus [53].

### Neural stem cell behaviour

The distinct role of CPs in both the control and production of CSF suggests this structure regulates neural stem cell behaviour—given their direct contact with CSF—and thereby neurogenesis and brain development [54, 55]. Indeed, there is evidence that CP-CSF signalling via the OTX2 homeoprotein is involved in the modulation of developmental neurogenesis and anxiety related behaviour [2, 56–62]. The specific mechanisms by which the CPs modulate brain development both in terms of the CPs as well as specific aspects of their functions (e.g., transfer of blood-borne molecules or secretion of a particular molecule) needs to be investigated. Further detailed studies of CP impact on different brain regions and developmental time-points would enhance our understanding of this process during brain development.

### A port of entry for immune cells into the brain parenchyma

Immune cells (or leukocytes) can access the brain parenchyma via the CP and circulate within the CSF [63]. Specifically, leukocytes can pass from the tightly regulated brain microvascular endothelium (i.e., the pia matter and the glia limitans) into the CP stroma and may then penetrate the CPE to enter the CSF. In this regard, an elegant study by Kertser and colleagues found that inducing severe stress in mice via fear conditioning (an established model for investigating post-traumatic stress disorder, PTSD) had a detrimental effect on immunosurveillance within CSF (viz., reduced influx of leukocytes to the CSF)—a mechanism that was linked to glucocorticoid signalling at the brain-immune interface [64]. Assessment of isolated CP tissue from the brain ventricles of mice following stress induction revealed lower mRNA levels of genes encoding for immune cell trafficking molecules including *Ccl2*, *Icam1* and *Cxc10* [64]. Notably, the authors of this study found that modulation of glucocorticoid receptor signalling either systemically or locally via genetic knockdown at the CP—of mice exposed to severe stress—facilitated the recruitment of GATA3 and FOXP3 regulatory T cells to the brain parenchyma, a process posited to attenuate the PTSD-like behaviour such as reduced exploratory drive [64]. In another recent study it was found that the CP serves as a conduit through which inflammatory state can be established in the developing brain in response to maternal immune activation—via exposure to the viral mimetic polyriboinosinic-polyribocytidilic acid in pregnant mice [65]. This study revealed that prenatal inflammatory exposure can drive a pro-inflammatory CSF signature and an accumulation of CP macrophages at particular ‘hotspots’ (viz., areas where the CP barrier integrity was disrupted such as the distal tips of CP villi) in the developing brain—a process triggered by elevated chemotactic CCL2-CCR2 signalling at the embryonic CP-CSF interface [65]. The authors state that the aberrant macrophage accumulation observed at the ventricular zone of the developing cerebral cortex of animals pre-exposed to immune activation during pregnancy is reflective of an increased level of CCL2 in the CSF that originates from the CP which may play an indirect role in the recruitment of CCR2-expressing phagocytic macrophages into the brain parenchyma [65]. These findings are particularly interesting from a neuropathological standpoint given that CSF from individuals with autism exhibit hallmarks of neuroinflammation including increased levels of CCL2 expression [66, 67].

The CP also plays a role in gut microbiota-immune interactions [21]. Notably, gut bacteria can modulate both gut-resident immune cells and brain-resident immune cells, with evidence indicating that activation of the immune system in the gut and in the brain are implicated in responses to neuroinflammation, brain injury, as well as changes in neurogenesis and plasticity [68, 69]. Interestingly, certain proteins involved in maintaining gut barrier integrity (e.g., Claudin-5) have the same role in the brain, such that gut-brain disruptions may occur via a shared pathological mechanism [70]. Another noteworthy observation is that the neurons that are found in the CP are similar to those observed in the enteric nerve bundles [71].

A growing stream of evidence has linked alterations in gut microbiome composition to the anomalies in CSF observed in neuropsychiatric disorders [72]. In this regard, a study conducted by Gorlé and colleagues found a link between CP inflammation and a disruption of the epithelial blood-CSF barrier upon *Helicobacter suis (H. suis)* infection [21]. These changes were accompanied by leakage of the gastrointestinal barrier and low-grade systemic inflammation, suggesting that *H. suis*-evoked gastrointestinal permeability and subsequent peripheral inflammation induces changes in brain homeostasis via changes in blood-CSF barrier integrity [21]. Interestingly, recent evidence revealed a CP vascular barrier that is modulated by the Wnt signalling pathway in response to intestinal inflammation via bacteria-derived lipopolysaccharide [73]. The same study found a deficit in short-term memory and anxiety-like behaviour in a model of genetically driven closure of CPE cells, suggesting that CP vascular closure may be associated with cognitive deficits—an aspect that may reflect a deregulated gut–brain vascular axis [73].

### Circadian rhythms

Circadian rhythms are a fundamental biological phenomena in almost all organisms, and control not only rest/activity rhythms but also a variety of other physiological functions such as hormone secretion, blood pressure, and body temperature regulation [74]. The molecular rhythm-generating mechanism is thought to rely on a feedback loop involving positively and negatively acting transcription factors [75]. At the core of the loop are the transcription factors CLOCK and BMAL1 that activate *Per1*, *Per2*, *Cry1*, and *Cry2* genes whose protein products PER and CRY repress their own transcription [75]. Animal studies found that clock genes are expressed in the CP [76], with expression levels of *Bmal1, Per1, Per2* and *Cry2* fluctuating throughout the day [77]. Recently, Myung and co-workers reported that the CP has a rigid rhythm of clock gene expression. They also proposed that the CP can regulate the suprachiasmatic nucleus through CSF circulation to finely tune circadian rhythms [18], a process linked to rhythmic metabolite clearance—via the glymphatic system (supported by AQP4)—according to the time of day [78]. In a study by Yamaguchi and colleagues, CP expression of *Per1*, *Per2*, and *Bmal1* genes in the lateral and fourth ventricles was modulated in a circadian manner and may control CSF secretion [79]. Other evidence has shown that glucocorticoids can reset the circadian clock in the CP via period genes. For instance, dexamethasone induced shifts of the CP clock may be mediated via PKA-ERK1/2 signalling. These results provide the first set of evidence that rhythmicity of glucocorticoid release can entrain the CP clock to assist with fine-tuning the brain in accordance with the time of day [80].

## The neuropathological role of the choroid plexus in neuropsychiatric and neurodevelopmental disorders

The structural and functional implications of the CP in neuropsychiatric and neurodevelopmental disorders have been neglected for many years [81]. However, in recent years the CP has gained attention as a neuroanatomical structure linked to brain diseases ranging from pain syndrome [82], microcephaly (via Zika virus disease) [83] and gliomas [84] to neurodevelopmental and neuropsychiatric disorders [85, 86]. In fact, since 2019 there have been 55 articles published in PubMed on this topic with an increasing trend over the past 20 years. In this section, we highlight the association between CP and brain diseases.

### Schizophrenia

CP abnormalities in schizophrenia are described as early as 1921 demonstrating morphological changes to the CPE, vascular endothelium, as well as hypersecretion [87, 88]. Several case reports reported on CP abnormalities being associated with mood, psychosis, and cognitive dysfunction [89–91]. Neuroimaging studies observed an association between CP calcification and psychosis symptoms and brain structural changes [92–94]. Also, transcriptomic analysis of the CP in schizophrenia was shown to have an upregulation of immune and inflammation genes, which significantly correlated with disease status and greater levels of cortisol, resistin, C-reactive protein, tissue inhibitor of metalloproteinase 1, matrix metallopeptidase 9 (MMP-9), and pro-inflammatory markers in the serum and frontal cortex in the same individuals [95]. These studies laid the foundation for subsequent studies enhancing the understanding of CP morphology and function in schizophrenia.

Our group previously reported CP volume enlargement in a large sample of patients with schizophrenia, schizoaffective, and bipolar disorder with psychosis compared to both their first-degree relatives and healthy controls [86]. Greater CP volume was independent of diagnostic categorization. First-degree relatives had intermediate CP volume compared to healthy controls and psychosis patients, as well as showing that CP volume was heritable [86]. Additionally, larger CP volume was associated with worse overall cognition, particularly in verbal fluency, attention and speed of information processing, but no correlations with clinical measures were identified. A link between CP volume and brain structure was also established, showing that greater CP volume is related to smaller gray matter and subcortical volume, larger ventricular volume, and lower white matter microstructure. A connection between higher peripheral levels of interleukin-6 and greater CP volume was also made, suggesting that inflammation may play a role in CP structural changes observed in psychosis. These findings were replicated in a first-episode schizophrenia (FES) study by Zhou et al., where they found greater CP volume in FES, which was correlated with higher allostatic load (indexed by subclinical cardiovascular, metabolic, neuroendocrine, and immune markers) [96]. The results from these two neuroimaging studies are promising, which taken together with the expanding interest of the CP in psychiatry and neurology [86, 96, 97], adds to the importance of ensuring accurate segmentation of the CP with more reliable and accurate tools [81].

Consistent with the neuroimaging findings above a recent study reported a larger CP volume in schizophrenia patients with orofacial tardive dyskinesia (TD) [98]. This study revealed that orofacial TD in schizophrenia may stem from elevated N-methyl-D-aspartate receptor antibody levels that are mediated by a disrupted CP. Thus, it is posited that CP volume could represent a sensitive structural biomarker for studies on the treatment and prevention of brain-periphery interaction abnormalities in orofacial TD [98].

### SARS-Cov-2 (COVID-19)

The CP has gained significant attention since the advent of the severe acute respiratory syndrome coronavirus 2 (SARS-Cov-2), commonly referred to as the COVID-19 pandemic. While the virus primarily impacts the respiratory system, neurological complications, including cerebrovascular injury, encephalopathy, neuropsychiatric and neurocognitive disorders have also been described [99]. Viral genetic material has been identified in the brain and CSF of patients with COVID-19 alongside neurological symptoms [100–102], but the pathophysiological mechanism still remains to be determined. Using CP organoids derived from induced pluripotent stem cells it was determined that SARS-CoV-2 sparsely impacts neurons and astrocytes, but selectively targets CPE cells and demonstrated an inflammatory phenotype resulting in viral-induced brain dysfunction [103]. This work was expanded upon to show that apolipoprotein- and ACE2-expressing CPE cells were primarily impacted leading to leakage across the blood-CSF barrier permitting the entry of pathogens, immune cells, and cytokines [104]. In addition to ACE2 (critical for SARS-CoV-2 attachment) being highly expressed in the CP and thalamus, ACE2 was also found to be expressed in excitatory/inhibitory neurons, astrocytes, oligodendrocytes, and endothelial cells located in the middle temporal and posterior cingulate cortex, some in the hippocampus, but none in the prefrontal cortex [105]. Despite direct effects on the CP, there are indirect effects of SARS-CoV-2 on endothelial cells and neurons, which are proposed to be mediated by systemic inflammatory- and/or immune-hyper response to infection [106]. To this point, a landmark study examining single-nucleus transcriptomes from the frontal cortex and CP of 14 control individuals and 8 patients with COVID-19, was not able to find any molecular traces of SARS-CoV-2 in the brain [107]. However, the authors demonstrate that CPE cells sense and relay peripheral inflammation into the brain, which may include T cell infiltration. Furthermore, connections are made between COVID-19 mediated pathological effects on microglia and astrocyte populations which have previously been reported in human neurodegenerative disease [108, 109]. Additionally, COVID-19 related effects on synaptic signalling in upper-layer excitatory neurons and other cell types were linked to chronic brain disorders associated with cognitive deficits including schizophrenia, autism and depression [107, 110]. Despite these important findings much still remains unknown about the method for neuroinvasion and what potential implications this might have on the development of neurocognitive and neuropsychiatric disorders, in particular as it relates to long COVID symptoms [111].

### Autism spectrum disorder

Similar to schizophrenia, there is a growing interest in understanding CP morphology in autism. The expansion of cranial ultrasounds led to the identification of minor abnormalities in neonates, such as subependymal pseudocysts, frontal horn cysts, or CP cysts. In a large sample of neonates receiving cranial ultrasound testing one week after birth, these patients were prospectively followed and received repeated neurodevelopmental assessments between the ages of 5 and 15. The authors found that while CP and frontal horn cysts were not associated with autism risk, subependymal cysts were associated with developmental delay in 5.5% of children and the odds ratios for developing autistic spectrum disorder was 28.54 [112]. In a large retrospective study of autism and neurotypical patients, the authors identified increased ventricular volumes among patients with autism and extended these findings to the CP [113]. This group also found that larger CP volume was associated with greater ventricle volumes in autism and neurotypical groups. Lastly, a texture-based analysis was performed of the CP using Laplacian-of-Gaussian filter, and the authors found that patients with autism had abnormalities in the spatial distribution of the CP compared to neurotypical individuals [114]. These observations are promising, but a direct causal relationship between CP volume or spatial distribution and ventricular volume remain to be established.

### Cerebral folate deficiency

Cerebral folate deficiency (CFD) syndrome is a neuropsychiatric condition associated with low CSF 5-methyltetrahydrofolate (MTHF) and normal folate in the blood [115]. This syndrome can result from autoantibodies, FOLR-1 mutations [116], mitochondrial dysfunction, and abnormal folate metabolism [116, 117]. The most common cause of CFD is serum blocking type autoantibodies against the folate receptor-α (FRα), which attaches to the basal side of the CPE [118]. This process leads to an inhibition of MTHF transport across the CP. The clinical presentation of CFD varies based on neurodevelopmental periods when folate deficiency takes place [116]. For example, FRα antibodies in either parent increases the risk of infantile autism in offspring. Infantile-onset CFD (4-6 months after birth) can present with autism, neurological sequelae, and spastic ataxia [119, 120], while developing CFD later can present as progressive dystonia or schizophrenia during adolescence [121]. In a study of schizophrenia patients resistant to standard of care treatment, 83% (15/18) of patients had positive serum FRα autoantibodies and 6 had low CSF folate levels. Seven patients received folinic acid over 6-months which resulted in positive and negative symptom improvement [115]. CFD syndromes are an example of how CP can be implicated in neurodevelopment or neuropsychiatric disorders, which reminds clinicians to adequately screen, diagnose and treat patients that may have a reversible cause of their clinical presentation.

## The choroid plexus as a treatment avenue for neurodevelopmental and neuropsychiatric disorders

The high metabolic activity (i.e. large mitochondrial content in CPE cells) and well developed extracellular matrix (ECM) of the CP provide important neuropathological implications for when the CP is altered. Notably, the CP can be induced to elicit neuroinflammatory responses in the presence of peripheral insults [122]. Furthermore, under chronic, unpredictable stress, the levels of ECM degrading enzymes—such as MMP-9—in the CP has been shown to be elevated [123]. This MMP-9 elevation has been linked to a release of pro-inflammatory cytokines due to nuclear factor-kappa B activation, which can trigger oxidative stress via free radical formation, resulting in damages to DNA, proteins, and lipids of neurons and other cell types in the CP. Conversely, neuroinflammation can increase the secretion of MMP-9 via microglia activation, leading to further oxidative damages, hence completing a vicious cycle between neuroinflammatory processes and oxidative stress [124, 125]. In particular, evidence from an animal model of infection revealed an upregulation of MMP-9 in the CP resulting in a reduction of Claudin-5, implying that infection caused tight junction breakdown. Notably, Claudin-5 was elevated in the CSF consistent with a disruption of the blood-CSF barrier. This disruption was attenuated by treatment with the MMP inhibitor GM6001 [126]. Although highly speculative, neuroinflammatory injury to the CP can compromise OTX2 production resulting in critical period anomalies with implications for mental illness. Thus, taken together, the CP emerges as a nexus of vulnerability in neuropsychiatric and neurodevelopmental disorders.

Understanding the mechanisms by which homeostatic disturbance at the CP–CSF interface is involved in neurodevelopmental and neuropsychiatric disorders can give new insights into therapeutic strategies. Delivery of therapeutic agents to the CNS is challenged by the barriers in place to regulate brain homeostasis. This is especially true for protein therapeutics. Targeting the CP barrier may be a surrogate brain delivery strategy to circumvent the BBB. However, as noted above for CFD the treatment would involve providing additional folate to overcome the FRα autoantibodies or FLOR-1 mutations [116]. Heterogenous cell populations located at the CP provide diverse functions in regulating the exchange of material within the ventricular space. In this regard, receptor-mediated transcytosis may be a promising mechanism to deliver protein therapeutics across the tight junctions formed by CPE cells. However, CSF flow and other barriers formed by ependymal cells and perivascular spaces should also be considered for evaluation of protein therapeutic disposition. It follows that further elucidation of the receptor–ligand co-localization and trafficking studies are needed to provide direct evidence in CPE cell transport systems. Further development of inducible knockout models targeting CPE cell transporters are also needed to better understand CP protein handling.

More recently, exosomes—a type of extracellular vesicle that contain constituents (e.g., protein, DNA, and RNA) of the cells that secrete them—have been recognized as potential therapeutic conveyors for brain-related diseases given their known role in the modulation of transcription, neurogenesis, plasticity, and neuroinflammation, each of which are affected in neuropsychiatric and neurodevelopmental disorders [127]. In this context, CP discharges exosomes into systemic circulation or CSF and CSF bulk flow promotes exosome distribution throughout the brain with potential therapeutic possibilities for improving CP neuron communication [128]. As such, barrier-generated vesicles, their composition and distribution deserve further investigation. Alternative approaches aimed towards the CP as a treatment avenue for CNS disorders include gene [129], transplantation [130], stem cell [131] and protein/peptide-based therapies [20] in addition to CP-epithelial drug targeting strategies [132]. With a better understanding of the physiological processes and comprehensive experimental techniques, the CPs have the potential to be targeted as a promising approach to treat specific neurodevelopmental and neuropsychiatric disorders.

## Conclusion

The unique anatomical localization of the CP directly influences brain function through CSF mediated transport of secreted proteins such as cytokines and hormones that in turn regulate neuronal signalling, brain plasticity and cognition. The recent discovery of CSF clearance routes, namely glymphatic efflux and the meningeal lymphatic network are of particular significance given the high metabolic rate and exquisite sensitivity of neurons and glia to alterations in their extracellular environment. It follows that understanding how the CP modulates CSF homeostasis including the entrance, maintenance, and exit of immune populations, extracellular proteins and micronutrients will provide an important framework to understand how perturbations in these key steps have an impact on brain development and are disrupted in neurodevelopmental and neuropsychiatric disease. In this context, the use of recently developed technologies such as iDISCO that enable high resolution imaging of structures deep within the brain—including the CP [133, 134]—will prove integral to better understanding aspects of CP physiology and their role in disease pathogenesis. Greater understanding of CP structure and function can be achieved using brain organoids. Notably, recent studies using brain organoids and human post-mortem tissue have linked SARS-Cov-2 (COVID-19) to a disruption of the CP with potential implications for neuropsychiatric complications [104, 107]. Thus, developing deeper knowledge of the CP-CSF interface will lead to novel routes and mechanisms for treatment of brain disorders. In this respect, further research is needed to unravel the mechanisms through which the CP might play a detrimental role in the development of neuropsychiatric disorders [135], which in turn, may open new therapeutic avenues to treat these conditions.
